# Retraction Note: Clinical application of contrast enhanced ultrasound to diagnose benign prostatic hyperplasia

**DOI:** 10.1186/s13000-015-0240-1

**Published:** 2015-07-02

**Authors:** Jingfang Shi, Xiaohua Yin, Rong Xu, Yingchun Wang, Lin Jin, Weiwei Gao

**Affiliations:** Department of Ultrasound, Jiading Center Hospital, Shanghai, 201800 China; Department of Radiology, Jiading Center Hospital, No.1 Chengbei Road, Jiading District, Shanghai 201800 China

## Retraction

The Publisher and Editor regretfully retract this article [1] because the peer-review process was inappropriately influenced and compromised. As a result, the scientific integrity of the article cannot be guaranteed. A systematic and detailed investigation suggests that a third party was involved in supplying fabricated details of potential peer reviewers for a large number of manuscripts submitted to different journals. In accordance with recommendations from COPE we have retracted all affected published articles, including this one. It was not possible to determine beyond doubt that the authors of this particular article were aware of any third party attempts to manipulate peer review of their manuscript.

### Update posted {2^nd^ July, 2015}

BioMed Central has been informed of the outcome of a thorough investigation by the institution. That investigation found that the authors of this article [1] intended to purchase language editing services for their manuscript only and did not participate in influencing the peer review process. The institution has taken further steps to educate their researchers regarding best practice in research and publication ethics.
